# Effects of gemcitabine on APE/ref-1 endonuclease activity in pancreatic cancer cells, and the therapeutic potential of antisense oligonucleotides

**DOI:** 10.1038/sj.bjc.6602080

**Published:** 2004-08-17

**Authors:** J P Lau, K L Weatherdon, V Skalski, D W Hedley

**Affiliations:** 1 Division of Experimental Therapeutics, Ontario Cancer Institute/Princess Margaret Hospital, 610 University Avenue, Toronto, Ontario, Canada M5G 2M9; 2 Department of Medical Biophysics, University of Toronto, 610 University Avenue, Toronto, Ontario, Canada M5G 2M9; 3 Department of Medical Oncology and Hematology, Ontario Cancer Institute/Princess Margaret Hospital, 610 University Avenue, Toronto, Ontario, Canada M5G 2M9

**Keywords:** base excision repair, antisense, APE/ref-1, gemcitabine, pancreatic cancer, drug resistance

## Abstract

Apurinic/apyrimidinic endonuclease (APE) is a key enzyme involved in DNA base excision repair (BER) that is often expressed at elevated levels in human cancers. Pancreatic cancer cells treated with the nucleoside analogue gemcitabine (2′, 2′-difluoro-2′deoxycytidine) showed increases in APE/redox effector factor (ref-1) protein levels (approximately two-fold for Panc-1 and six-fold for MiaPaCa-2), with corresponding increases in endonuclease activity. These results suggested that the activation of APE/ref-1 might be an adaptive response that contributes to gemcitabine resistance by facilitating BER. To test this hypothesis, we examined the effects of disrupting APE/ref-1 using antisense on gemcitabine toxicity. Antisense oligonucleotides decreased protein levels three-fold in MiaPaCa-2 and five-fold in Panc-1 in comparison to controls, associated with reduced endonuclease activity. Combination treatments with antisense oligonucleotides and gemcitabine partially suppressed the increase in APE/ref-1 activity seen in cells exposed to gemcitabine alone. While clonogenic assays showed only slight decreases in colony formation in cells treated with either antisense oligonucleotides or gemcitabine alone, the combination with APE/ref-1 antisense resulted in a 2-log enhancement of gemcitabine toxicity in Panc-1 cells. Overall these findings suggest that APE/ref-1 plays a significant role in gemcitabine resistance in some pancreatic cancer cells, and support the further investigation of novel treatments that target this protein.

There are over 20 000 apurinic/apyrimidinic (AP) sites formed per cell per day making it one of the most common forms of DNA damage (Evans *et al*, 2000; Kelley and Parsons, 2001). Abasic sites can arise due to spontaneous and chemically initiated hydrolysis through various conditions such as exposure to ionising radiation, oxidative stress or cytotoxic agents. The persistence of these abasic sites in DNA can lead to a halt in DNA replication, mutations and an overall loss in genetic stability.

The DNA base excision repair (BER) pathway is involved in maintaining DNA integrity through the removal and replacement of abasic sites (Evans *et al*, 2000). Once the damaged base has been identified by DNA glycosylases, the base is removed leaving an abasic site, which is hydrolysed by the apurinic/apyrimidinic endonuclease (APE) at position 5′ to the abasic site, leaving a 3′ hydroxyl and an abasic deoxyribose-5-phosphate. The abasic site is removed by deoxyribose phosphatase, then DNA polymerase *β* and DNA ligase I or DNA ligase III/XRCCI replaces the nucleotide and closes the gaps between the newly synthesised nucleotide and the strand. Aside from BER, the APE protein is also involved in maintaining various transcription factors in their active reduced states such as AP-1, NF-*κ*B, HIF-1 and p53 (Hirota *et al*, 1997; Ema *et al*, 1999; Ueno *et al*, 1999). Therefore, APE is also commonly referred to as redox effector factor (ref-1) protein.

APE/ref-1 levels have been shown to be elevated in various types of cancers such as cervical, prostate, ovarian and germ cell tumours (Xu *et al*, 1997; Herring *et al*, 1998; Moore *et al*, 2000; Kelley *et al*, 2001; Robertson *et al*, 2001). Previous studies have shown that a decrease in APE/ref-1 protein renders mammalian cells sensitive to methyl methanesulphonate and hydrogen peroxide (Walker *et al*, 1994), while radioresistance of human cervical tumours directly correlates with increased APE/ref-1 expression (Herring *et al*, 1998). Following exposure to irradiation and oxidative stress, tumour cells showed elevated APE/ref-1 protein levels (Herring *et al*, 1998; Ramana *et al*, 1998; Silber *et al*, 2002), while overexpression of APE/ref-1 in testicular cancer resulted in bleomycin resistance (Robertson *et al*, 2001).

Attempts to create APE/ref-1 knockout mice (APEX−/−) were embryonically lethal, suggesting that APE/ref-1 is crucial for embryonic development (Xanthoudakis *et al*, 1996; Ludwig *et al*, 1998). Heterozygous APE/ref-1 mice (APEX+/−) were viable but abnormally sensitive to oxidative stress and prone to cancer development (Meira *et al*, 2001).

Pancreatic ductal adenocarcinoma is the fifth leading cause of cancer death in North America. It is highly resistant to conventional cytotoxic agents, and is almost 100% lethal. Currently, the only active agent appears to be a chain terminator, gemcitabine (2′, 2′-difluoro-2′deoxycytidine), and there is an urgent need to develop new and more effective treatment (Abbruzzese, 2002; Haller, 2003). In this paper, we demonstrate that treatment of pancreatic cancer cells with gemcitabine significantly induces APE/ref-1 protein expression and endonuclease activity, and that the suppression of APE/ref-1 activity by antisense oligonucleotides produces a large increase in chemosensitivity in gemcitabine-treated Panc-1 cells.

## MATERIALS AND METHODS

### Cell lines and culture

The MiaPaCa-2 and Panc-1 human pancreatic cell lines (American Type Culture Collection, Rockville, MD, USA) were maintained in Dulbecco's medium with L-glutamine, 0.1 mg ml^−1^ kanamycin and 10% foetal bovine serum (FBS) (GibcoBRL, Burlington, Ontario, Canada). Cells were grown at 37°C and 5% CO_2_ in a humidified atmosphere. Cell cultures were passaged routinely once a week and re-established from frozen stock every 2 months.

### Western blot analysis

Protein was extracted from cells treated with lysis buffer containing protease inhibitor cocktail tablets (Roche Canada, Mississauga, Ontario, Canada), 50 mM HEPES (pH=8.0), 10% glycerol, 1% Triton X-100, 150 mM NaCl, 1 mM EDTA, 1.5 mM MgCl_2_, 100 mM NaF, 10 mM NaP_2_O_7_·H_2_O) and centrifuged at 14 000 r.p.m. at 4°C for 10 min. Samples were loaded in a 12% polyacrylamide gel, with 1 × running buffer and allowed to run in a gel chamber (Bio-Rad Laboratories, Mississauga, Ontario, Canada). Following transfer to nitrocellulose membranes, these membranes were incubated in 1 : 1000 primary APE/ref-1 antibody (Novus Biologicals, Littleton, CO, USA) and probed with 1 : 2000 goat anti-mouse monoclonal antibody (Amersham Biosciences, Baie d'Urfé, Quebec, Canada). Membranes were exposed to ECL and developed on film (Kodak, New Haven, CT, USA), then stained and destained with amido black to determine protein loading.

### Flow cytometry analysis

**Figure 1:**
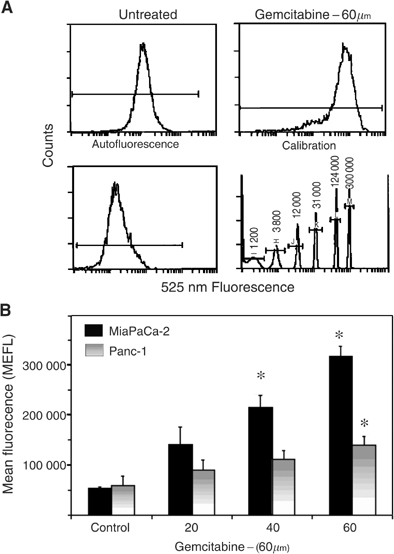
APE/ref-1 protein levels in control and gemcitabine-treated MiaPaCa and Panc-1 monolayers. (**A**) Representative flow cytometry data for MiaPaCa cells treated with 60 *μ*M gemcitabine for 48 h compared to untreated control, autofluorescence background and calibration beads. Gated on light scatter to exclude dead cells. (**B**) APE/ref-1 protein levels in MiaPaCa and Panc-1 controls and cells treated with 20, 40 and 60  *μ*m gemcitabine for 48 h, expressed as mean equivalent fluorescein (MEFL) values obtained from the calibration beads. Bars represent means from three separate experiments±s.e. Stars indicate results that are statistically significant (*P*<0.05) with respect to the nontreated samples.

Cells were adjusted to 1 × 10^6^ cells ml^−1^ and fixed with 2% formaldehyde for 10 min at 37°C, followed by 90% ice-cold methanol for 30 min. Prior to antibody staining, cells were rinsed with 2 ml phosphate-buffered saline (PBS) containing 4% FBS. Based on a preliminary dilution curve showing that this concentration is saturating, 1 : 1000 dilution of APE/ref-1 antibody (Novus Biologicals, Littleton, CO, USA) was added to the pellet for 30 min at room temperature, then washed twice in PBS plus 4% FBS. A goat-anti-mouse FITC-labelled secondary antibody (Caltag Laboratories, Burlingame, CA, USA) was added to the pellet for 15 min at room temperature, and the sample washed twice with PBS plus 4% FBS and resuspended in 1 ml of PBS plus 4% FBS. Samples were analysed by flow cytometry (Epics Elite; Beckman-Coulter, Miami, FL, USA) using 488 nm excitation and collecting through a 525 nm bandpass filter. To standardise the flow cytometry results, calibration particles (Rainbow Beads; Spherotech Inc., Libertyville, IL, USA) were measured in each sample run. A calibration curve was generated based on the mean fluorescence values for the Rainbow Beads, as illustrated in Figure 1

, and used to convert the mean fluorescence of the samples into the number of FITC molecules per cell.

### Gemcitabine treatment

Gemcitabine was obtained from Eli Lilly & Co (Indianapolis, IN, USA). Exponentially growing cells were plated at ∼10^5^ cells ml^−1^ in T-25 cm^2^ flasks, and drug added at desired concentrations to the medium and incubated for 24 or 48 h. After treatment, cells were washed, harvested and counted for the preparation of cell lysates, or fixed for flow cytometry analysis.

### Transfection of antisense oligonucleotides

Two antisense oligonucleotides were synthesised (ACGT, Toronto, Ontario, Canada) based on sequences recently published by Silber *et al* (2002). One antisense oligonucleotide targeted the translational start site of APE/ref-1 mRNA, while the other oligonucleotide targeted the exon–intron junction of the pre-mRNA. Oligonucleotides synthesised in the sense direction were used as controls. The oligonucleotides were transfected into cells of 60–70% confluency. In one well of a 12-well plate, 250 nM of each of the oligonucleotides was added along with 12 *μ*l of Plus Reagent (Invitrogen, Burlington, Ontario, Canada) and Opti-MEM media (Invitrogen, Burlington, Ontario, Canada) adjusted to a total volume of 100 *μ*l and allowed to stand for 15 min at room temperature. In another well, 4 ml of Lipofectamine Reagent was added to 96 ml of Opti-MEM media and allowed to stand for 15 min. Precomplexed DNA was combined with the diluted Lipofectamine reagent and incubated at room temperature for 15 min. Growth medium was removed from flasks and DNA–Plus–Lipofectamine Reagent complexes containing 800 *μ*l of fresh Opti-MEM media was added and mixed gently. Cells were allowed to incubate at 37°C at 5% CO_2_ for 4 h. Opti-MEM was then removed and replaced with normal growth medium containing 250 nM of each of the antisense oligonucleotides.

In order to measure the uptake kinetics for each oligonucleotide by flow cytometry and to determine the effects of lipofectamine, the translational start site targeting antisense oligonucleotide was labelled with FITC and the exon–intron targeting antisense oligonucleotide was labelled with Cy5.5. The flow cytometry technique was similar to that for APE/ref-1 protein measurement, with the additional use of a 633 nm HeNe laser to excite Cy5.5.

### Purification of APE/ref-1 protein

Human APE/ref-1 wild-type protein was overproduced using the pGEX-3X (Pharmacia Biotech, Uppsala, Sweden) system utilising the *Bam*HI 5′ and *Eco*RI 3′ restriction sites, obtained from Dr Mark Kelley (Indiana University). The transformations were performed using One Shot® TOP 10 Competent Cells (Invitrogen, Burlington, Ontario, Canada) with transformed *Escherichia coli* cells spread on Luria–Bertani (LB) agar plates containing ampicillin. Colonies found to contain the transformed plasmid were placed in LB broth containing 100 *μ*g ml^−1^ ampicillin and incubated overnight at 37°C. For batch purification of protein, 200 *μ*l of glutathione sepharose 4B slurry (Amersham Biosciences, Baie d'Urfé, Quebec, Canada) was equilibriated and the bacterial sonicate allowed to incubate at room temperature for 30 min. To sediment the gel, glutathione elution buffer was added, mixed gently and then centrifuged at 500 **g** for 5 min. The supernatant was then dialysed for 2 h at 4°C in 100 volumes of GST. The concentration of the GST-tagged protein was determined by assuming 1 absorbance unit at 280 nm was equal to 0.5 mg ml^−1^. The purified APE/ref-1 protein was stored in aliquots at −20° and used as the positive control in the endonuclease assay.

### Endonuclease assay

A 26-mer oligonucleotide (IDT Technologies, Coralville, IA, USA) containing a tetrahydrofuran (F) residue at position 15 was used, as described by Kelley *et al* (2001) and Kelley and Parsons (2001). Following ^32^P labelling, the oligonucleotide was purified using a G25 column and then annealed to a complementary oligonucleotide. Based on 2.0 × 10^5^ cells lysed, 1 *μ*l of total cell extract was added to 10 *μ*l reaction volume containing 5000 c.p.m. of labelled double-stranded F oligonucleotide in 50 mM HEPES, 50 mM KCl, 10 mM MgCl_2_, 2 mM DTT, 1 *μ*g ml^−1^ bovine serum albumin and 0.05% Triton X-100, adjusted to pH 7.5. Reactions were allowed to proceed for 5 min in a 37°C water bath and stopped by adding 4 *μ*l of 96% formamide, 10 mM EDTA and bromophenol blue. The positive control was the purified APE/ref-1 protein (0.5–2.0 *μ*g), and the negative control consisted of cells treated with the BER inhibitor methoxyamine hydrochloride 33 mM (Sigma-Aldrich Canada Ltd, Oakville, Ontario, Canada) mixed in buffer containing 50 mM KPO_4_, (pH 7.1), and exposed to double-stranded oligonucleotide for 15 min in a 37°C water bath prior to adding lysate. Samples were separated with a 15% polyacrylamide gel containing 7 M urea and exposed to a Molecular Dynamics Phosphoimager to measure the intensities of the substrate and product bands, and to film overnight for visualisation. Calculation of the fraction of product formed was based on Fraction of product formed=Area product/(Area product+Area substrate).

### Clonogenic assay

Aliquots of 10^5^ cells were taken from cell suspensions treated with antisense or sense oligonucleotides and/or gemcitabine and diluted serially in growth medium post-treatment, plated in six-well cell culture plates (Gibco BRL, Burlington, Ontario, Canada) at final cell densities of 10^4^, 10^3^ and 10^2^ cells well^−1^. After 7 and 12 days of incubation at 37°C in 95% air and 5% CO_2_ for MiaPaCa-2 and Panc-1, respectively, medium was removed and plates allowed to air dry. Colonies were counter-stained with methylene blue in 70% ethanol for 5 min and counted.

### Statistical analysis

Statistical analyses were performed with Jandel Sigma Stat software, Version 2.0. For comparisons between treatments, the analysis of variance test of equal variance or Kruskal–Wallis was applied with the Dunnett's method. A *P*-value of *α*=0.05 indicated statistical significance.

## RESULTS

### Gemcitabine effects on APE/ref-1 protein

Preliminary experiments showed that both cell lines maintained >90% viability, based on flow cytometric assessment of light scatter and propidium iodide exclusion, following exposure to up to 60 *μ*M gemcitabine for up to 48 h. To examine whether gemcitabine was able to induce APE/ref-1 levels, MiaPaCa-2 and Panc-1 cells were treated with a range of gemcitabine concentrations for 24 or 48 h, and APE/ref-1 protein levels measured using Western blot or flow cytometry. Preliminary experiments showed that these two techniques gave comparable results. However, the flow cytometry method was more rapid and easier to standardise and was therefore used for most experiments. As shown in Figure 1, the two cell lines showed similar basal levels of APE/ref-1, and each showed a gemcitabine dose-dependent increase following 48 h of continuous treatment. Relative to untreated controls, MiaPaCa-2 cells treated with 40 and 60 *μ*M of gemcitabine showed four- and six-fold increases in APE/ref-1 protein expression (*P*=0.012 and 0.003, respectively), while Panc-1 cells treated with 60 *μ*M of gemcitabine showed a two-fold increase (*P*=0.047). Smaller increases in APE/ref-1 were also seen after 24 h treatment with gemcitabine in both cell lines (data not shown).

### *In vitro* assessment of APE/ref-1 endonuclease activity of gemcitabine-treated cells

**Figure 2:**
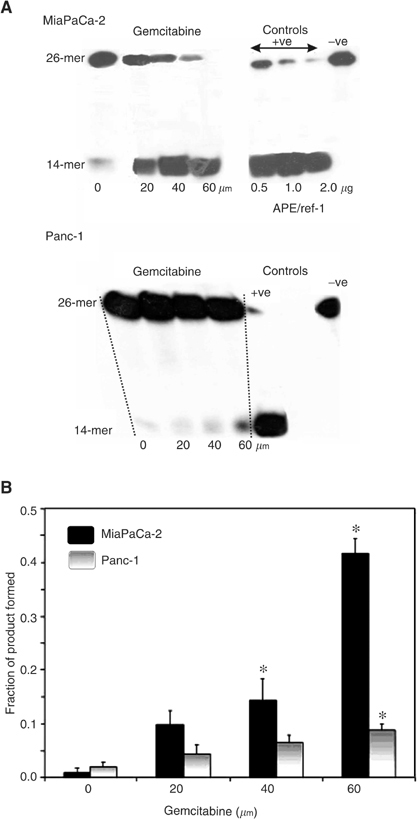
Endonuclease activity present in gemcitabine-treated cells. (**A**) Film images of 26-mer substrate *vs* 14-mer product in MiaPaCa and Panc-1 cells after various doses of gemcitabine for 48 h. Positive controls consisted of purified recombinant APE/ref-1 protein and negative controls included the BER inhibitor methoxyamine. (**B**) Range of gemcitabine concentrations *vs* the fraction of 14-mer product formed. Stars indicate results that are statistically significant (*P*<0.05) with respect to the nontreated samples. The concentration of gemcitabine is expressed as *μ*M. All values are means of three independent experiments±s.e.

**Figure 3:**
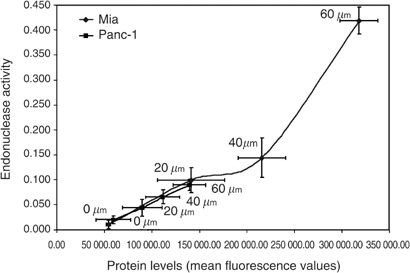
Relationship between APE/ref-1 protein level *vs* endonuclease activity postgemcitabine exposure. MiaPaCa (diamonds) and Panc-1 (squares) cells were treated with various doses of gemcitabine for 48 h. Samples from the same flask were collected and assessed using both flow cytometry and endonuclease assay. Points represent mean values from three separate experiments and error bars are standard error of the mean; *r*=0.951 (MiaPaCa) and 0.999 (Panc-1).

To examine the effects on endonuclease activity, MiaPaCa-2 and Panc-1 cells were treated with various concentrations of gemcitabine for 48 h, then lysed and assayed for catalytic activity. More 14-mer product was formed, with a corresponding decrease in 26-mer substrate, at increasing concentrations of gemcitabine (Figure 2A

). Both cell lines showed dose-dependent increases in endonuclease activity following treatment with gemcitabine (Figure 2B). Using 60 *μ*M of gemcitabine, the levels were six-fold greater (*P*<0.05) than control for MiaPaCa-2 and two-fold greater (*P*<0.05) than control for Panc-1. MiaPaCa-2 and Panc-1 monolayers were treated with gemcitabine for 48 h and samples for protein expression and endonuclease activity were taken from the same flask to determine the correlation between endonuclease activity and APE/ref-1 protein expression. As shown in Figure 3

, these values were highly correlated in both MiaPaCa-2 (*r*=0.951) and Panc-1 (*r*=0.999).

### Uptake kinetics of fluorescently labelled antisense oligonucleotides

**Figure 4:**
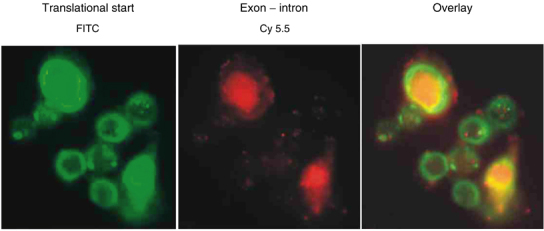
Subcellular localisation of fluorescence-labelled antisense oligonucleotides. That targeting the exon–intron junction of APE/ref-1 pre-mRNA is localised in the nucleus, whereas the translational start is cytoplasmic.

The antisense oligonucleotide targeting the translational start site of APE/ref-1 mRNA was labelled with FITC, while the other antisense oligonucleotide targeting the exon–intron junction of APE/ref-1 pre-mRNA was labelled with Cy5.5. Subcellular localisation of the antisense oligonucleotides was examined using fluorescence microscopy and the translational start site targeting antisense was found in the cytoplasm, while the exon–intron targeting antisense oligonucleotide was located in the nucleus (Figure 4

). Preliminary experiments using flow cytometry showed very low levels of fluorescently labelled oligonucleotide uptake in the control cells relative to lipofectamine-treated cells (data not shown). With lipofectamine, the uptake of both antisense oligonucleotides appeared to saturate at concentrations above 250 nM. Based on the flow cytometry calibration beads, the mean fluorescence values for cells transfected at this concentration correspond to approximately 4 × 10^5^ oligonucleotides cell^−1^ for the FITC-labelled construct. A concentration of 250 nM of each antisense oligonucleotide was chosen for future experiments.

### Effects of APE/ref-1 antisense on protein expression and endonuclease activity

**Figure 5:**
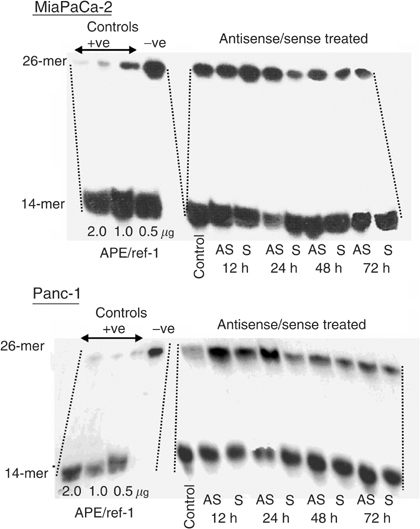
Endonuclease activity of MiaPaCa and Panc-1 cells treated with antisense oligonucleotides. The MiaPaCa and Panc-1 cell lines were exposed to antisense or sense oligonucleotides for various time points. Cell lysates were collected and analysed using the endonuclease assay. C – control; AS – antisense; S – sense.

Both cell lines were transfected with antisense or sense oligonucleotides and APE/ref-1 protein levels were analysed 12, 24, 48 and 72 h post-transfection. Using both Western blot and flow cytometry, there were statistically significant decreases in protein levels detected after 24 h of transfection with MiaPaCa-2 decreasing three-fold (*P*<0.001) and Panc-1 decreasing four-fold (*P*<0.001). After 48 h post-transfection, the APE/ref-1 levels had substantially recovered. The level of endonuclease activity was assessed in cell lysates, obtained from the same flasks as the samples taken for protein measurements. As illustrated in Figure 5

, a reduction in the amount of 14-mer product formed *vs* substrate was seen 24 h post-transfection, with recovery of endonuclease activity at 48 h, consistent with the decrease in protein levels. Treatment with oligonucleotides that were synthesised in the sense direction had no significant effects on APE/ref-1 protein levels or endonuclease activity.

### APE/ref-1 protein and endonuclease activity in gemcitabine-treated cells: effects of antisense

**Figure 6:**
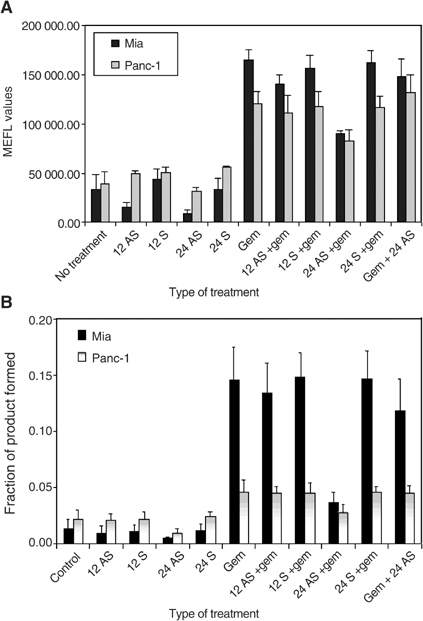
APE/ref-1 protein levels (**A**) and endonuclease activity (**B**) post-treatment with antisense/sense oligonucleotide±gemcitabine. (**A**) Protein levels. MiaPaCa (dark bars) and Panc-1 (light bars) cells were treated with antisense or sense oligonucleotides±20 *μ*M gemcitabine. APE/ref-1 protein levels were accessed at 12 or 24 h post-transfection of oligonucleotides or 24 h post-treatment with gemcitabine. As indicated, some samples were treated with gemcitabine prior to transfection with antisense oligonucleotides to determine whether the sequence of treatment affected outcome. Bars represent three individual experiments, with error bars representing standard error of the mean. AS – antisense; S – sense; Gem – gemcitabine.

To examine if APE/ref-1 antisense was able to suppress the increases in protein levels and endonuclease activity seen following treatment with gemcitabine, experiments involving the treatment of cells with both antisense oligonucleotides and gemcitabine were performed. Both MiaPaCa-2 and Panc-1 cell lines were transfected with antisense or sense oligonucleotides for 12 and 24 h with some samples receiving an additional 24 h treatment of 20 *μ*M of gemcitabine. There was a decrease in APE/ref-1 induction when the cells were transfected with antisense prior to gemcitabine exposure, relative to the samples treated with gemcitabine alone or gemcitabine plus oligonucleotides synthesised in the sense direction (Figure 6A

). Similarly, endonuclease activity was lower in the cells transfected with antisense oligonucleotides prior to gemcitabine treatment *vs* the cells treated with gemcitabine alone (Figure 6B).

### *In vitro* sensitivity of APE/ref-1 antisense-treated Panc-1 and MiaPaCa-2 cells to gemcitabine

**Figure 7:**
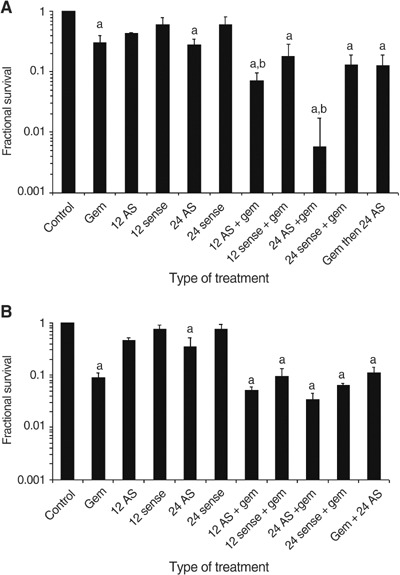
Clonogenic survival of (**A**) Panc-1 and (**B**) MiaPaCa cells treated with gemcitabine alone, with antisense (AS) or sense oligonucleotide for 12 or 24 h or the combination. Bars represent at least three individual experiments, with standard error of the mean indicated by the error bars. ^a^Statistically significant (*P*<0.05) compared to control samples. ^b^Statistically significant (*P*<0.05) with respect to the gemcitabine-only treated samples.

Colony-forming assays were performed on both cell lines treated with antisense or sense oligonucleotides and gemcitabine. The MiaPaCa-2 cells were found to be more gemcitabine sensitive than Panc-1 in the clonogenic assay in preliminary experiments, and the dose was therefore reduced to 5 *μ*M for MiaPaCa-2. Samples were also treated with gemcitabine for 24 h prior to transfection with antisense to determine whether the order of administration influenced clonogenic survival. As shown in Figure 7

, lower numbers of colonies were formed by MiaPaCa-2 and Panc-1 cells treated with antisense alone, gemcitabine alone or the combination, when compared to cells treated with sense oligonucleotides or untreated controls (*P*<0.05). Gemcitabine-treated Panc-1 cell lines showed a 2-log decrease in colony formation when the cells were pretreated for 24 h with antisense oligonucleotides compared to cells treated with gemcitabine alone or in combination with the sense oligonucleotides (Figure 7A). Despite the suppression of APE/ref-1 activity in MiaPaCa-2 cells treated with antisense, the effects on gemcitabine sensitivity relative to the sense construct were much smaller and not statistically significant (Figure 7B).

## DISCUSSION

In this study, we show that gemcitabine is capable of inducing APE/ref-1 levels in two pancreatic cancer cell lines, MiaPaCa-2 and Panc-1. The increase in APE/ref-1 protein levels was closely correlated with an increase in endonuclease activity, suggesting that APE/ref-1 is the predominant endonuclease enzyme in these cells. These results suggest the existence of a cellular response that might facilitate the repair of gemcitabine-induced DNA damage, potentially thereby enhancing drug resistance. To investigate this further, we examined the effects of antisense oligonucleotides against APE/ref-1 mRNA, using constructs recently described by Silber *et al* (2002) and treatment schedules based on initial experiments that measured the uptake of fluorescence-labelled oligonucleotides. These antisense oligonucleotides were able to suppress gemcitabine-induced APE/ref-1 activity when given prior to but not following gemcitabine treatment. We avoided exposure to gemcitabine immediately following treatment with lipofectamine, and the data shown in Figure 6 were obtained 36 and 48 h following antisense treatment. Since the maximum effect of the antisense oligonucleotides was seen at 24 h, the effects on APE/ref-1 in combination with gemcitabine might be underestimated. Panc-1 cells, which were highly resistant to gemcitabine based on clonogenic assay, showed a large enhancement in gemcitabine toxicity when treated with APE/ref-1 antisense. This finding of enhanced sensitivity to a DNA-damaging agent is similar to but greater than earlier results obtained by Walker *et al* (1994) using stable transfectants of the full-length APE/ref-1 antisense and the more recent results obtained by Silber *et al* (2002) using phosphorothioate antisense oligonucleotides. However, although MiaPaCa-2 cells showed similar responses to those seen in Panc-1 following treatment with gemcitabine and antisense in terms of APE/ref-1 protein levels and endonuclease activity, the sensitisation to gemcitabine by the antisense was small and not statistically significant relative to the sense control.

The effects of gemcitabine on APE/ref-1 protein induction seen in the present study are greater than those previously reported involving oxidative stress or *γ*-irradiation (Herring *et al*, 1998; Ramana *et al*, 1998; Silber *et al*, 2002). However, to our knowledge this study is the first to address the effects of deoxynucleoside analogues on APE/ref-1 activity. It should be noted that although the concentrations of gemcitabine used in these experiments approximate the peak plasma levels achieved using standard dose schedules, the continuous exposure for 48 h exceeds that seen in cancer patients. Therefore, it remains to be determined if APE/ref-1 activity is increased during treatment with gemcitabine in the clinic. In preliminary experiments, we have found that another deoxycytidine analogue, cytosine arabinoside (ara-C), is able to elevate APE/ref-1 levels in OCI/AML-2 leukaemia cells (not shown), suggesting that this might be a widespread response to treatment with deoxynucleoside analogues.

Previous studies have demonstrated elevated levels of another BER protein, DNA ligase I, in MiaPaCa-2 cells treated with ara-C and gemcitabine (Gandhi *et al*, 1996; Gandhi *et al*, 1997). The enhancement of DNA ligase I protein due to ara-C exposure was not accompanied by increased DNA synthesis or polymerase activity. DNA ligase I levels returned to normal as the drug-treated cells resumed DNA synthesis, thus strongly suggesting the increased DNA ligase I levels were involved in repair of DNA damage caused by ara-C, rather than DNA replication (Sun *et al*, 2002a; 2002b). Also, it has been previously shown that the antisense oligonucleotides used in the present paper are able to reduce APE/ref-1 protein and endonuclease activity in human glioma cells, with concurrent reduction in resistance towards the alkylating agents methyl methanesulphonate and temozolomide (Silber *et al*, 2002). Another study examining the effects of methoxyamine, an inhibitor of BER through binding of abasic sites, found that this enhanced sensitivity to temozolomide in human colon cancer xenografts (Liu *et al*, 2002). Taken together, these studies suggest that the BER pathway plays an important role in cancer chemotherapy resistance, and that suppression of BER might therefore result in increased chemosensitivity.

The enhancement of APE/ref-1 activity following gemcitabine treatment suggests that this might be playing a role in the repair of DNA damage, although we recognise that it is unclear if APE/ref-1 acts on misincorporation of gemcitabine into DNA. The present series of experiments does not establish that the sensitising effects of APE/ref-1 antisense is due to suppression of its BER activity, since APE/ref-1 also acts as a redox regulator of several transcription factors including NF-*κ*B and AP1 that can promote cell survival responses (Hirota *et al*, 1997; Arrigo, 1999; Ueno *et al*, 1999; Moos *et al*, 2003). For example, the activation of NF-*κ*B can occur during treatment with cancer chemotherapy and is believed to promote drug resistance (Wang *et al*, 1999; Baldwin, 2001; Arlt *et al*, 2003). The increases in APE/ref-1 seen in pancreas cancer cells during treatment with gemcitabine might augment this NF-*κ*B response, additional to any effects on BER. The BER and redox effector activities of APE/ref-1 can be differentially regulated by phosphate modifications, and each function involves complex interactions with other cellular proteins (Yacoub *et al*, 1997; Evans *et al*, 2000; Hsieh *et al*, 2001). The striking difference observed between the sensitisation of Panc-1 and MiaPaCa-2 cells to gemcitabine following APE/ref-1 antisense treatment is likely to be explained on the basis of differences involving these elements, and further work is needed in this area.

Phosphorothioate antisense olignonucleotides have been developed as therapeutic agents targeting a number of genes involved in cancer development, and several are currently undergoing clinical trial (O'Dwyer *et al*, 1999; Morris *et al*, 2002). Using APE/ref-1 antisense, we obtained a two decade decrease in the clonogenic survival of highly resistant Panc-1 cells treated with gemcitabine. Although it is not known if clinically achievable gemcitabine levels can upregulate APE/ref-1 activity in cancer patients, or if selective targeting of APE/ref-1 would enhance the therapeutic index of gemcitabine in pancreas cancer patients, they warrant further investigation including *in vivo* testing in xenograft models, examination of other pancreas cancer cell lines and elucidation of the underlying mechanisms. This work is currently in progress in our laboratory.
